# A Seropositive Nodular Rheumatoid Polyarthritis without Arthritis: Does It Exist?

**DOI:** 10.1155/2012/983985

**Published:** 2012-04-17

**Authors:** Fatma Ben Fredj Ismail, Amel Rezgui, Monia Karmani, Olfa Ben Abdallah, Samira Azzebi, Chedia Laouani Kechrid

**Affiliations:** Research Unit for Clinical Geriatrics, Department of Internal Medicine, Sahloul Hospital, 01/UR/08-12, 4054 Sousse, Tunisia

## Abstract

The rheumatoid polyarthritis is the most frequent chronic polyarthritis. It affects essentially the woman between 40 and 60 years. Rheumatic subcutaneous nodules and tenosynovitis are usually associated with seropositive symptomatic rheumatoid polyarthritis. It is, however, rare that they constitute the essential clinical expression of the disease. In this case, it makes dispute another exceptional form of rheumatoid arthritis such as rheumatoid nodulosis. A 60-year-old woman was hospitalized for tumefaction of the dorsal face of the right hand evolving two months before. The clinical examination found subcutaneous nodules from which the exploration ended in rheumatoid nodules with tenosynovitis. The evolution after four years was favourable under corticosteroid therapy, methotrexate, and colchicine.

## 1. Introduction

The rheumatoid polyarthritis (RP) is the most frequent shape of the chronic polyarthritis, affecting joints provided with a synovial membrane. It is characterized by often bilateral and symmetric arthritis of several articular groups touching preferentially wrists and hands, evolving by pushes towards the deformation and the destruction of affected joints articulations. It gets essentially women between 40 and 60 years. The rheumatic subcutaneous nodules and the tenosynovitis are usually associated in seropositive RP. It is, however, rare that they constitute the essential clinical expression of the disease. We report a particular observation by its mode of revelation and its clinical evolution. 

## 2. Observation

**Figure 1:**
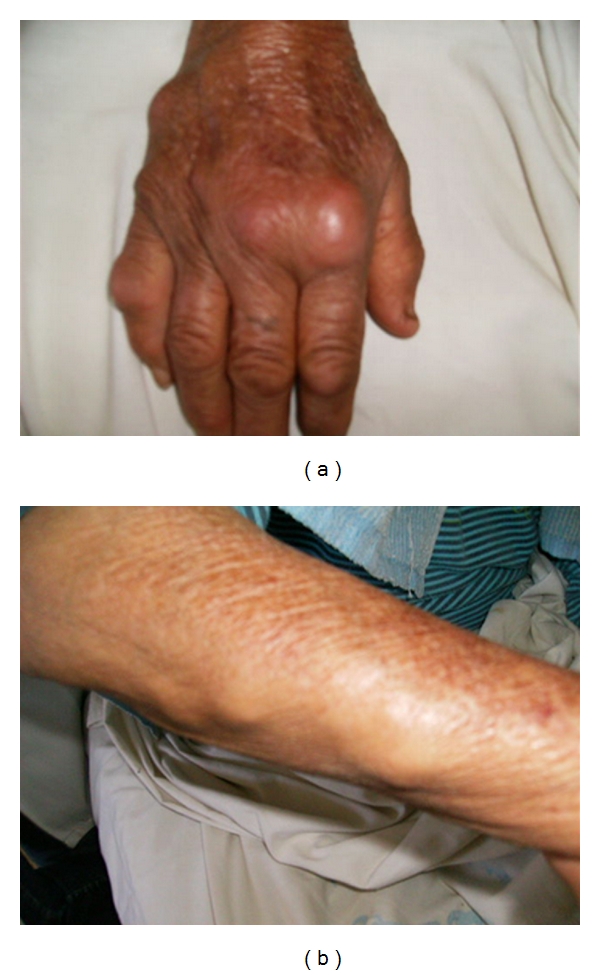
Multiple mobile painful nodules on irregular surface of the dorsal face of the right hand as well as the front homolateral arm.

60-year-old woman with history of asthma under a long-term inhaled corticoids, consulted for tumefaction of the dorsal face of the right hand evolving for two months without triggering factor neither fever nor change of the general state. The interrogation found a notion of ancient arthralgia of large and small joints without arthritis and without any other systematic sign. The physical examination noted the presence of multiple mobile painful nodules on irregular surface of the dorsal face of the right hand as well as the front homolateral arm (Figures 1(a) and 1(b)), making two at four centimeters of main line with pain in the articular mobilization without arthritis nor limitation of movements. Biologically, there was an inflammatory syndrome and negative rheumatoid factor. Besides, antibodies anti-cyclic citrullines peptides (anti-CCP) were positive to 42 UI/mL and the antinuclear antibodies were negative. To the radiography of hands, we noted a demineralization in bands without osteolysis. An echography and a magnetic resonnance imaging of mild parts then a MRI found a tissular, vasculated and heterogeneous mass of four centimeters, of the right hand, evoking a tenosynovitis. This mass was associated with several other nodules, and the biopsy the biggest revealed its rheumatoid nature in the anatomopathological examination. A treatment with colchicine, general and local corticosteroid therapy in infiltration, and methotrexate was then established. The evolution at seventeen months was marked by a disappearance of rheumatoid nodules and the persistence of a little tumor corresponding to the tenosynovitis.

## 3. Discussion

The rheumatoid polyarthritis (RP) is a chronic disease of complex and multifactorial determinism, unpredictable evolution, and sometimes difficult positive and differential diagnosis. The ancient ACR criteria of RP contain four articular clinical criteria on seven and remain debatable [1]. Rheumatoid nodules and factors give probably less to discussion [1]. In the new classification published in 2010, rheumatic subcutaneous nodules are no longer major factors for the diagnosis of rheumatoid polyarthritis [2].The definition of the typical radiographic modifications of RP is probably very debatable. Besides, a radiographic reading made by a rheumatologist knowing the clinical symptomatology influences the interpretation, in particular as regards the demineralization [1]. At our case report, the rheumatoid polyarthritis was essentially retained on the presence during the rheumatoid exploration of a nodule confirmed by the anatomopathology and the positive anti-CCP antibodies. The sex and age (60-year-old woman), the tenosynovitis of the right hand, the notion of chronic arthralgia, and the clinicobiologic evolution under treatment supported our diagnosis.

Classically, numerous arguments plead for an early diagnosis of RP. Effectively, osteocartilaginous destructions appear early in the evolution of the disease (10 to 15% of the RP have erosions after three months of evolution, 30% after one year, 70% after three years, and 95% after six years), and they are irreversible [3]. At our patient, the diagnosis of RP was able to be retained in spite of the absence of arthritis, thanks to the rather specific extra-articular appearances of the disease. The absence of true arthritis in our observation could be due to a low systematic passage of corticoids inhaled in the long term by the patient. Our case report may be another observation of a different subset of rheumatoid arthritis such as rheumatoid nodulosis witch was described first by Bywaters in 1949, then individualized by Ginsberg in 1975 [4, 5].

The rheumatoid factor is mostly absent during the first six months of evolution of RP. However, the presence of anti-CCP antibodies has a 50% of sensibility in a beginner RP, but especially a high specificity (97%) [6]. It was the case in our observation.

A polyarthritis miming one PR can reveal other chronic inflammatory rheumatisms or even a neoplasm particularly a malignant lymphoma [7]. At our case report, the sex, the age, the interrogation, the clinical examination, and the biological explorations eliminated other causes of arthralgia with subcutaneous nodules.

The imaging by magnetic resonance can improve the diagnosis of tenosynovitis during RP [8]. In our observation, it was in favour of a tenosynovitis and it eliminated the diagnosis of a malignant tumor and an infectious collection.

## 4. Conclusion

The extra-articular appearances of the rheumatoid polyarthritis must not be underestimated especially since they can constitute—even rarely—the essential clinical expression of the disease.
